# Microbial Enzymatic Synthesis of Amikacin Analogs With Antibacterial Activity Against Multidrug-Resistant Pathogens

**DOI:** 10.3389/fmicb.2021.725916

**Published:** 2021-08-27

**Authors:** Yeon Hee Ban, Myoung Chong Song, Joong Ho Jeong, Min Seok Kwun, Chang Rae Kim, Hwi So Ryu, Eunji Kim, Je Won Park, Dong Gun Lee, Yeo Joon Yoon

**Affiliations:** ^1^College of Pharmacy, Natural Products Research Institute, Seoul National University, Seoul, South Korea; ^2^School of Life Sciences, BK21 FOUR KNU Creative BioResearch Group, College of Natural Sciences, Kyungpook National University, Daegu, South Korea; ^3^Department of Integrated Biomedical and Life Sciences, Korea University, Seoul, South Korea

**Keywords:** amikacin analogs, microbial enzymatic synthesis, multidrug-resistant pathogens, antibacterial activity, cytotoxicity

## Abstract

With the constant emergence of multidrug-resistant gram-negative bacteria, interest in the development of new aminoglycoside (AG) antibiotics for clinical use has increased. The regioselective modification of AG scaffolds could be an efficient approach for the development of new antibiotics with improved therapeutic potency. We enzymatically synthesized three amikacin analogs containing structural modifications in the amino groups and evaluated their antibacterial activity and cytotoxicity. Among them, 6′-*N*-acyl-3^″^-*N*-methylated analogs showed improved antibacterial activity against the multidrug-resistant gram-negative bacteria tested, while exhibiting reduced *in vitro* nephrotoxicity compared to amikacin. This study demonstrated that the modifications of the 6′-amino group as well as the 3^″^-amino group have noteworthy advantages for circumventing the AG-resistance mechanism. The regiospecific enzymatic modification could be exploited to develop novel antibacterial agents with improved pharmacological potential.

## Introduction

Aminoglycosides (AGs), produced from a group of soil microorganism actinomycetes, are one of the oldest classes of antibiotics. They have strong antibacterial activity against a broad range of gram-negative and gram-positive pathogens due to interference with protein biosynthesis by acting on the bacterial ribosome. However, as with other antibiotics, antimicrobial resistance due to the excessive use of AGs has emerged and spread rapidly (Sana and Monack, 2016; Serio et al., 2018). AG resistance can arise from various mechanisms, such as the enzymatic modification of the chemical structure of AG, modification of the target site via methylation or chromosomal mutation, and increased cellular drug efflux by small molecule transporters. Of these mechanisms, the direct modification of the AG structure by AG-modifying enzymes (AMEs), which include *N*-acetyltransferases (AACs), *O*-adenyltransferases (ANTs), and *O*-phosphotransferases (APHs), is the major mechanism underlying antibiotic resistance (Becker and Cooper, 2013; Krause et al., 2016; Serio et al., 2018).

**FIGURE 1:**
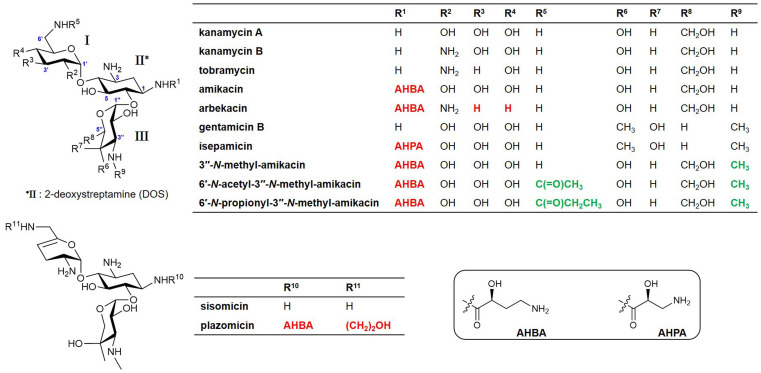
Structures of natural aminoglycosides (AGs) and their semisynthetic analogs currently used in the clinic and new amikacin analogs developed in this study. Red colors represent the chemically modified features of the semisynthetic AGs and green colors represent the functional groups formed by enzymatic synthesis in this study.

A native AG, butirosin, has an (*S*)-4-amino-2-hydroxybutyric acid (AHBA) substituent at the C1-amine, which is introduced by the acyltransferase BtrH and its partner BtrG. This AHBA moiety is a natural defensive structural element that can improve the clinical activity against AG-resistant pathogens by protecting AG from several common AMEs (Llewellyn et al., 2007). This has led to substantial efforts to chemically develop semisynthetic AGs to evade clinically relevant AMEs and improve their anti-infective therapeutic potential. For example, amikacin, isepamicin, and arbekacin have been synthesized by adding 1-*N*-AHBA side chains to kanamycin A, gentamicin B, and kanamycin B, respectively (Figure 1). The latest approved semisynthetic AG, plazomicin, is also a sisomicin derivative that has an AHBA side chain and a hydroxyethyl group at the C1 and C6′-amine positions, respectively (Figure 1; Becker and Cooper, 2013; Krause et al., 2016; Serio et al., 2018).

Although chemical modification approaches for the development of semisynthetic AGs have been successful, there are limitations due to the structural complexity of AGs (Chandrika and Garneau-Tsodikova, 2018). Hence, other complementary approaches to develop new agents with higher potency against AG-resistant pathogens and an improved safety profile are warranted. Microbial enzymatic synthesis could become an effective alternative to the chemical method for manufacturing AGs in clinical use and developing new AGs for the post-antibiotic era. It has been reported that the substrate tolerance of BtrH and BtrG, responsible for the addition of an AHBA moiety to the butirosin structure, was exploited to regiospecifically introduce an AHBA side chain onto the non-native AGs (Llewellyn et al., 2007; Llewellyn and Spencer, 2008). Other enzymatic approaches to generate *N*-acylated AGs using AMEs have also been reported. Two AACs, AAC(3) and AAC(6′)-APH(2^″^), exhibited promiscuity toward a number of AGs, as well as diverse acyl-CoAs, resulting in the generation of novel mono- and hetero-di-*N*-acylated AGs (Green et al., 2010; Porter et al., 2010; Tsitovich et al., 2010; Holbrook and Garneau-Tsodikova, 2016; Ban et al., 2020a). Additionally, the relaxed substrate specificity of AG biosynthetic enzymes can be exploited for the efficient regiospecific modification of AG structures (Park et al., 2013; Ban et al., 2020b). For example, GenN involved in gentamicin biosynthesis has been previously shown to catalyze 3^″^-*N*-methylation in both kanamycin B and tobramycin, indicating its potential to generate novel AGs (Huang et al., 2015; Bury et al., 2017).

The previous structure-activity relationship results have suggested that selective substitution of one or more amino groups at the C1, C6′, and C3^″^-positions of AGs could provide new, more potent antibiotics (Maeda et al., 1968; Chandrika and Garneau-Tsodikova, 2016; Santana et al., 2016; Ban et al., 2020a). Here, we report the enzymatic synthesis of new amikacin analogs, appending a methyl group at the C3^″^-amine position alone or together with different acyl chains at the C6′-amine, using 6′-*N*-acetyltransferase AAC(6′)-APH(2^″^) and 3^″^-*N*-methyltransferase GenN. The antibacterial activity and cytotoxicity of the newly synthesized amikacin analogs were evaluated, in order to investigate whether these modifications had a determining effect on the bioactivity. This study clearly demonstrated that regiospecific enzymatic modifications could be an efficient alternative to the chemical method for the generation of novel AG analogs, and the simultaneous modification of C6′ and C3^″^-amine positions is critical for the development of new AG antibiotics with an improved pharmacological potential.

## Materials and Methods

### The Microbial Enzymatic Synthesis of Amikacin Analogs

The *genN* and *aac(6*′*)-aph(2*^″^*)* genes derived from *Micromonospora echinospora* (GenBank accession no. AJ628149.4) and *Staphylococcus aureus* (GenBank accession no. NC_002774), respectively, were codon-optimized for expression in *Escherichia coli* and synthesized from DNA 2.0 (Menlo Park, CA, United States). To obtain the recombinant proteins with a histidine-tag, the synthesized *genN* DNA fragment was cloned into pJExpress404 (DNA 2.0, Menlo Park, CA, United States), whereas the *aac(6′)-aph(2^″^)* DNA fragment was cloned into pET15b (Novagen, Madison, WI, United States). For the expression and purification of GenN and AAC(6′)-APH(2^″^), the expression plasmids were individually introduced into *E. coli* BL21(DE3). The recombinant strains were grown in LB medium supplemented with 50 μg/mL ampicillin. Each liter of culture was inoculated with 10 mL of overnight starter culture, and the resulting mixture was grown at 37°C to an OD_600_ of 0.6. Overexpression of the desired proteins was induced by 0.5 mM IPTG at 37°C for another 3 h.

Cells were harvested by centrifugation (2 min at 14,981 × *g*), resuspended in lysis buffer (300 mM NaCl, 10 mM imidazole, 50 mM sodium phosphate, pH 8.0), and then lysed by sonication for 5 min using a 1 s on/1 s off cycle. The lysate was clarified by centrifugation (30 min at 44,496 × *g*), and the clarified cell lysate was passed through a column of Ni-nitrilotriacetic acid (NTA) agarose (Qiagen, Valencia, California, United States). After washing the column with washing buffer (300 mM NaCl, 40 mM imidazole, 50 mM sodium phosphate, pH 8.0), histidine-tagged recombinant proteins were eluted with elution buffer (300 mM NaCl, 500 mM imidazole, 50 mM sodium phosphate, pH 8.0). Imidazole was removed from the purified recombinant protein solution using a PD10 column (GE Healthcare, Piscataway, NJ, United States) with Tris buffer (20 mM Tris–HCl, 250 mM NaCl, 10% glycerol, pH 7.9). The purified proteins were concentrated using an Amicon Ultracel 10 K molecular weight cut-off spin filter (Millipore, Bedford, MA, United States), and then stored at −80°C. Sodium dodecyl sulfate-PAGE (SDS-PAGE) analysis was employed to ascertain the purity of protein. Protein concentration was determined by the Bradford protein assay using bovine serum albumin as a standard.

The new amikacin analogs were formed from the amikacin by large-scale one-pot enzymatic reaction. To produce 3^″^-*N*-methyl-amikacin, purified GenN (500 μM) was incubated with 2 mM amikacin (MP Biomedicals, LLC, Parc d’innovation, Illkirch, France) and 2 mM *S*-adenosyl-L-methionine (SAM) in 50 mM Tris–HCl (pH 7.5) at 30°C for 12 h. Two new 6′-*N*-acyl-3^″^-*N*-methylated amikacin analogs, 6′-*N*-acetyl-3^″^-*N*-methyl-amikacin and 6′-*N*-propionyl-3^″^-*N*-methyl-amikacin, were synthesized using a one-pot enzymatic reaction of AAC(6′)-APH(2^″^) and GenN. Purified AAC(6′)-APH(2^″^) and GenN (500 μM) were incubated with 2 mM amikacin, 2 mM acyl-CoAs (acetyl-CoA or propionyl-CoA; Sigma-Aldrich, St Louis, MO, United States), and 2 mM SAM in 50 mM Tris–HCl (pH 7.5) at 30°C for 12 h. The reaction was quenched with chloroform and centrifuged at 25,318 × *g* for 10 min. The supernatant containing the product of interest was extracted using an ODS solid phase extraction (SPE) cartridge, Bond Elut-C18 (Agilent Technologies, Santa Clara, CA, United States), reconstituted with water, and then analyzed using ultra-performance liquid chromatography-quadrupole and time-of-flight-high resolution-mass spectrometry (UPLC-qTOF-HR-MS), as previously described (Ban et al., 2020a). For the elucidation of structure and biological activity, approximately 5 mg of these new amikacin analogs were obtained from 5 mL of large-scale enzyme reactions.

### Structural Identification of Newly Synthesized Amikacin Analogs

The enzymatically synthesized samples were applied to a Sep-Pak^®^ C18 Vac cartridge (Waters Inc., Milford, MA, United States) and eluted with a heptafluorobutyric acid (HFBA) solution. The C18 cartridge was conditioned with 1% HFBA solution (elution with methanol (MeOH) 3 mL → 50% *aq.* MeOH (1% HFBA) 3 mL → 1% HFBA 3 mL), and then the supernatant containing 1% HFBA was loaded and eluted under the following conditions: 3 mL of 1% HFBA, 3 mL of 5% *aq.* MeOH (1% HFBA), 3 mL of 10% *aq.* MeOH (1% HFBA), and 3 mL of MeOH. The converted amikacin analogs were recovered from 10% *aq.* MeOH (1% HFBA) and 3 mL of MeOH, and concentrated *in vacuo*.

To purify the three converted products, each concentrated amikacin analog was refractionated by elution with a step gradient solvent system composed of MeOH and 1% HFBA solution (0, 1, 2, 5, 10, 20, and 100% MeOH in 1% HFBA solution). 3^″^-*N*-methyl-amikacin in 2–5% *aq*. MeOH fractions was collected as pure compound. 6′-*N*-acetyl-3^″^-*N*-methyl-amikacin and 6′-*N*-propionyl-3^″^-*N*-methyl-amikacin within 2–10% *aq*. MeOH fractions were collected as pure compounds.

The nuclear magnetic resonance (NMR) spectra of the newly synthesized amikacin analogs were obtained using a Bruker Avance 600 spectrometer (Billerica, MA, United States) operating at 600 MHz for ^1^H and 150 MHz for ^13^C nuclei. Samples for NMR analysis were prepared by dissolving each compound in 400 μL of D_2_O and placing the solutions in 5 mm NMR microtubes. All NMR data were processed using the Mnova software (Mestrelab Research S.L., Santiago de Compostela, Spain).

### Antibacterial Susceptibility Test

*Escherichia coli* (ATCC 25922) and *Pseudomonas aeruginosa* (ATCC 27853) were obtained from the American Type Culture Collection (ATCC). Five clinical multidrug-resistant (MDR) *P. aeruginosa* (MDRPA) isolates were obtained from patients at Kyungpook National University Hospital (KNUH), located in Daegu, South Korea. The isolates were identified using the API20NE kit (BioMerieux, Marcy-l′Etoile, Lyon, France) (Gurung et al., 2010). Antibiotic-resistant *E. coli* were provided by the National Biobank of KNUH, a member of the Korea Biobank Network-KNUH, obtained (with informed consent) under institutional review board (IRB)-approved protocols. Bacteria were grown in LB medium (BD, Franklin Lakes, NJ, United States) at 37°C.

Bacteria were cultured in LB medium, and the cell suspensions were adjusted to obtain standardized populations by measuring the turbidity with a spectrophotometer (DU530; Beckman, Fullerton, CA, United States). The bacterial strains at the exponential phase (1 × 10^6^ cells/mL) were inoculated, and 100 μL was dispensed per well of 96-well microtiter plates. Susceptibility tests were performed using a 2-fold standard broth microdilution of the test compounds, following the Clinical and Laboratory Standards Institute (CLSI) guidelines. After 18 h of incubation at 37°C, the minimum concentration required to prevent the growth of 90% of a given test organism was defined as the minimum inhibitory concentration (MIC_90_). Growth was assayed using a microtiter ELISA Reader (BioTek ELx800; BioTek Instruments Inc., Winooski, VT, United States) by monitoring the optical density at 600 nm (Choi and Lee, 2012).

### Cytotoxicity Assay Against Mammalian Renal Cell Lines

To examine the *in vitro* cytotoxicity of newly synthesized amikacin analogs, a series of MTT-based cell toxicity assays were carried out using three kidney-derived mammalian cell lines from ATCC [HEK-293 (human), LLC-PK1 (pig), and A-498 (human)] as previously described (Ban et al., 2019, 2020a). The concentration of half-maximal lethal dose for cells (LC_50_) was estimated by fitting concentration response curves to the data obtained from at least two independent experiments, using the SigmaPlot software package 10.0.1 (Systat Software Inc., San Jose, CA, United States). Data were expressed as means (*n* = 3) ± standard deviations and tested for significance using the paired or unpaired two-tailed *t*-test with analysis of variance as appropriate.

## Results and Discussion

### Microbial Enzymatic Synthesis of Amikacin Analogs Using AAC(6′)-APH(2^″^) and GenN Enzymes

**FIGURE 2:**
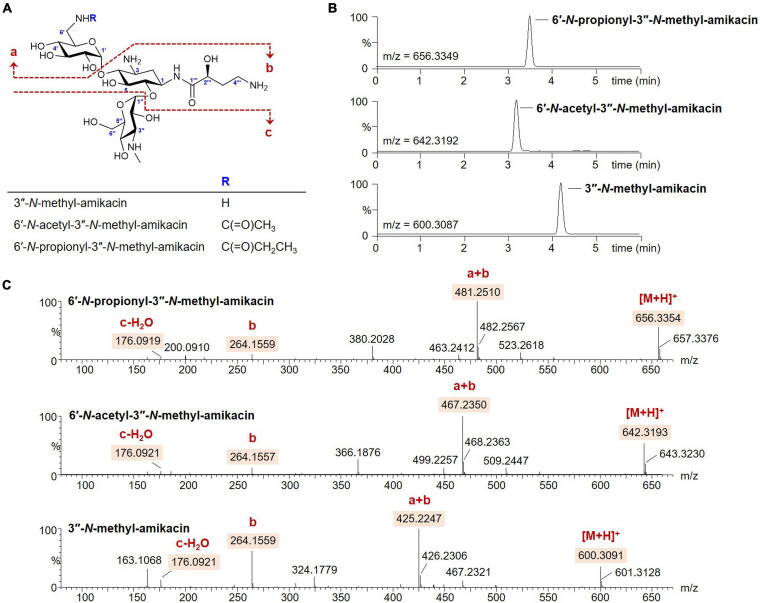
Enzymatic synthesis of the amikacin analogs. **(A)** Structures of the amikacin analogs produced by the microbial enzymatic synthesis. **(B)** UPLC-qTOF-HR-MS analysis of the amikacin analogs. Chromatograms are shown for selected 3^″^-*N*-methyl-amikacin (selected *m/z* 600.3087), 6′-*N*-acetyl-3^″^-*N*-methyl-amikacin (selected *m/z* 642.3192), and 6′-*N*-propionyl-3^″^-*N*-methyl-amikacin (selected *m/z* 656.3349). **(C)** MS/MS fragmentation patterns of the amikacin analogs.

The modification of 6′ or 3^″^-amino groups on AG scaffolds could be a promising approach to develop novel AG analogs with more robust pharmacological activity against resistant bacterial strains (Maeda et al., 1968; Santana et al., 2016). However, the desired chemical modification at a specific amine position on the AG structure remains challenging owing to its structural complexity (Chandrika and Garneau-Tsodikova, 2018). Therefore, we opted to exploit the enzymatic method for the generation of novel analogs modified at the two different amine positions. It has been reported that the substrate promiscuity of AAC(6′)-APH(2^″^) leads to diverse acylations at the C6′-amine position on the amikacin scaffold (Green et al., 2010; Holbrook and Garneau-Tsodikova, 2016). In contrast, the activity of GenN was confirmed only toward kanamycin B and tobramycin, which lack the AHBA side chain at the C1-amine compared to amikacin (Huang et al., 2015; Bury et al., 2017). The histidine-tagged 6′-*N*-AG acetyltransferase AAC(6′)-APH(2^″^) and 3^″^-*N*-methyltransferase GenN were expressed in *E. coli* and purified (Supplementary Figure 1), and we first examined the specificity of GenN toward amikacin. Amikacin was an excellent substrate for SAM-dependent methylation by GenN, and the formation of a new amikacin analog was detected using UPLC-qTOF-HR-MS at a conversion yield of approximately 100% (Figures 2A,B). Analysis of the MS fragmentation pattern confirmed that the methylation occurred specifically on the third ring [D-3-glucosamine, also known as kanosamine (Kan) moiety], consistent with the formation of 3^″^-*N*-methyl-amikacin (Figures 2A,C). This showed that GenN possesses broad substrate specificity to accept amikacin as an efficient substrate, despite the significant structural differences between amikacin and its natural substrate gentamicin: amikacin has a hexose as the third ring with 1-*N*-AHBA chain, while gentamicin congeners have a pentose as the third ring without an AHBA chain. Next, one-pot enzymatic reactions were attempted to simultaneously modify the 6′ and 3^″^-amino groups of amikacin. Upon incubation of amikacin with GenN and AAC(6′)-APH(2^″^) in the presence of acyl-CoAs, the formation of 6′-*N*-acyl-3^″^-*N*-methylated products was observed. Incubation with acetyl-CoA and propionyl-CoA as cosubstrates led to the formation of new UPLC peaks corresponding to 6′-*N*-acetyl-3^″^-*N*-methyl-amikacin and 6′-*N*-proprionyl-3^″^-*N*-methyl-amikacin, at conversion yields of approximately 100% in both cases (Figures 2A,B). The structures of these two amikacin derivatives were also predicted using MS fragmentation patterns (Figures 2A,C). Interestingly, the AAC(6′)-APH(2^″^) and GenN enzymes exhibited similar catalytic efficiencies for the additionally modified amikacin derivatives at the 3^″^-*N* or 6′-*N*-positions compared with amikacin. These results suggested that the activity of these regiospecific enzymes was not notably affected by the structural alteration of other sugar moieties on the AG scaffolds. Newly synthesized amikacin analogs were isolated from large-scale enzyme reactions, and their structures were confirmed with NMR and UPLC-qTOF- HR-MS.

### Structural Characterization of New Amikacin Analogs

The three amikacin analogs of 3^″^-*N*-methyl-amikacin, 6′-*N*-acetyl-3^″^-*N*-methyl-amikacin, and 6′-*N*-proprionyl-3^″^-*N*-methyl-amikacin were purified using a C18 sample preparation cartridge, and the structure of each compound was determined using HR-qTOF-MS and 600 MHz NMR analysis.

3^″^-*N*-methyl-amikacin was isolated as a white amorphous powder. The molecular formula of 3^″^-*N*-methyl-amikacin was found to be C_2__3_H_4__5_N_5_O_13_ (*m*/*z* 600.3091 [M + H]^+^, calculated for C_2__3_H_4__6_N_5_O_13_^+^, 600.3087) based on its HR-qTOF-MS data. The characteristic fragmentation pattern was observed at [M + H – 3-*N*-methylkanosamine (NMeKan)]^+^ 425.2247, [1-*N*-AHBA-2-deoxystreptamine (DOS) + H]^+^ 264.1559, [NMeKan + H − H_2_O]^+^ 176.0921, and [6-aminoglucose(aminoGlc) + H − H_2_O]^+^ 162.0748 by 4,6-glycosidic link cleavage (Figures 2A,C). The ^13^C NMR data (Supplementary Figure 2B and Table 1) confirmed the presence of 23 carbons. Comprehensive analysis of ^1^H, ^13^C, and HSQC NMR spectroscopic data of 3^″^-*N*-methyl-amikacin revealed the presence of a carbonyl signal (δ_C_ 175.5), two anomer signals (δ_H_ 5.42/δ_C_ 95.6; δ_H_ 5.07/δ_C_ 98.1), two *N*-methylene signals (δ_H_ 3.34 and 3.10/δ_C_ 40.2; δ_H_ 3.07/δ_C_ 36.9), three *N*-methine signals (δ_H_ 4.01/δ_C_ 48.8; δ_H_ 3.49/δ_C_ 47.7; δ_H_ 3.37/δ_C_ 61.0), an *N*-methyl signal (δ_H_ 2.71/δ_C_ 29.5), an *O*-methylene signal (δ_H_ 3.70/δ_C_ 59.6), 11 *O*-methine signals (δ_H_ 3.76/δ_C_ 79.2; δ_H_ 3.78/δ_C_ 66.2; δ_H_ 3.75/δ_C_ 80.1; δ_H_ 3.57/δ_C_ 70.7; δ_H_ 3.68/δ_C_ 72.3; δ_H_ 3.29/δ_C_ 70.7; δ_H_ 3.93/δ_C_ 68.7; δ_H_ 3.79/δ_C_ 72.4; δ_H_ 3.73/δ_C_ 63.5; δ_H_ 4.01/δ_C_ 72.1; δ_H_ 4.18/δ_C_ 69.5), and 2 methylene signals (δ_H_ 2.10 and 1.70/δ_C_ 30.3; δ_H_ 2.13 and 1.86/δ_C_ 30.7). This is the typical feature of an amikacin analog with a methyl group. The COSY correlation of H-3′′′ (δ_H_ 2.13/1.86) with H-2′′′ (δ_H_ 4.18) and H-4′′′ (δ_H_ 3.07), and HMBC correlation from H-1 (δ_H_ 3.96) and H-2′′′ (δ_H_ 4.18) to C-1′′′ (δ_C_ 175.5) revealed the AHBA at C-1 of DOS (Supplementary Figures 2C,E). The ^1^H-^13^C HMBC coupling correlation from H-1′ (δ_H_ 5.42) and H-1′′ (δ_H_ 5.07) to C-4 (δ_C_ 79.2) and C-6 (δ_C_ 80.1), respectively, showed a 4,6-glycosidic link of DOS with aminoGlc and Kan, respectively. The AHBA group, aminoGlc, and Kan on C-1, C-4, and C-6 of DOS including additional methyl group, respectively, were constructed using COSY and HMBC correlations (Supplementary Figures 2C,E). In the HMBC spectrum, an *N*-methyl proton (δ_H_ 2.71) to C-3^″^ (δ_C_ 61.0) of Kan showed a crosspeak indicating the attachment of a methyl group on the amine group at C-3^″^. Thus, the structure of 3^″^-*N*-methyl-amikacin was defined as a novel amikacin analog with an *N*-methyl on C-3^″^.

**TABLE 1:** ^1^H and ^13^C NMR data of novel amikacin analogs in D_2_O.

**No.**	**3^″^-*N*-methyl-amikacin**		**6′-*N*-acetyl-3^″^-*N*-methyl-amikacin**		**6′-*N*-propionyl-3^″^-*N*-methyl-amikacin**	
	**δ _C_**	**δ _H_ (*J* in Hz)**	**δ _C_**	**δ _H_ (*J* in Hz)**	**δ _C_**	**δ _H_ (*J* in Hz)**
1	48.8	4.01 (ddd, 9.6, 9.6, 3.6, 1H)	48.7	4.01 (ddd, 9.6, 9.6, 3.6, 1H)	48.7	4.01 (ddd, 9.6, 9.6, 3.6, 1H)
2	30.1	2.10 (ddd, 12.6, 3.6, 3.6, 1H)	30.2	2.11 (ddd, 12.6, 3.6, 3.6, 1H)	30.1	2.10 (ddd, 12.6, 3.6, 3.6, 1H)
		1.70 (ddd, 12.6, 9.6, 9.6, 1H)		1.67 (ddd, 12.6, 9.6, 9.6, 1H)		1.70 (ddd, 12.6, 9.6, 9.6, 1H)
3	47.7	3.49 (ddd, 9.6, 9.6, 3.6, 1H)	48.4	3.40 (ddd, 9.6, 9.6, 3.6, 1H)	48.4	3.40 (ddd, 9.6, 9.6, 3.6, 1H)
4	79.2	3.76 (dd, 9.6, 9.6, 1H)	79.2	3.73 (dd, 9.6, 9.6, 1H)	79.3	3.76 (dd, 9.6, 9.6, 1H)
5	66.2	3.78 (dd, 9.6, 9.6, 1H)	66.3	3.76 (dd, 9.6, 9.6, 1H)	66.3	3.77 (dd, 9.6, 9.6, 1H)
6	80.1	3.75 (dd, 9.6, 9.6, 1H)	80.1	3.68 (dd, 9.6, 9.6, 1H)	80.4	3.75 (dd, 9.6, 9.6, 1H)

1′	95.6	5.42 (d, 3.6, 1H)	97.9	5.42 (d, 3.6, 1H)	97.8	5.30 (d, 3.6, 1H)
2′	70.7	3.57 (dd, 9.6, 3.6, 1H)	70.7	3.57 (dd, 9.6, 3.6, 1H)	72.0	3.52 (dd, 9.6, 3.6, 1H)
3′	72.3	3.68 (dd, 9.6, 9.0, 1H)	72.3	3.68 (dd, 9.6, 9.0, 1H)	72.2	3.62 (dd, 9.6, 9.0, 1H)
4′	70.7	3.29 (dd, 9.0, 9.0, 1H)	70.7	3.29 (dd, 9.0, 9.0, 1H)	70.3	3.21 (dd, 9.0, 9.0, 1H)
5′	68.7	3.93 (ddd, 9.0, 6.6, 2.4, 1H)	68.7	3.73 (ddd, 9.0, 6.6, 2.4, 1H)	68.7	3.74 (ddd, 9.0, 6.6, 2.4, 1H)
6′	40.2	3.34 (dd, 13.2, 2.4, 1H)	39.6	3.34 (dd, 13.2, 2.4, 1H)	39.5	3.43 (dd, 13.2, 2.4, 1H)
		3.10 (dd, 13.2, 6.6, 1H)		3.10 (dd, 13.2, 6.6, 1H)		3.40 (dd, 13.2, 6.6, 1H)
7′	–	–	174.6	–	178.6	–
8′	–	–	21.8	1.90 (s, 3H)	29.0	2.16 (q, 7.2, 1H)
9′	–	–	–	–	8.2	1.01 (t, 7.2, 1H)

1^″^	98.1	5.07 (d, 3.6, 1H)	97.9	5.07 (d, 3.6, 1H)	97.9	5.07 (d, 3.6, 1H)
2^″^	72.4	3.79 (dd, 9.6, 3.6, 1H)	72.4	3.79 (dd, 9.6, 3.6, 1H)	73.0	3.75 (dd, 9.6, 3.6, 1H)
3^″^	61.0	3.37 (dd, 10.8, 9.6, 1H)	61.0	3.37 (dd, 10.8, 9.6, 1H)	61.1	3.37 (dd, 10.8, 9.6, 1H)
4^″^	63.5	3.73 (dd, 10.8, 9.6, 1H)	63.5	3.73 (dd, 10.8, 9.6, 1H)	63.5	3.71 (dd, 10.8, 9.6, 1H)
5^″^	72.1	4.01 (dt, 9.6, 3.0, 1H)	72.1	4.01 (dt, 9.6, 3.0, 1H)	72.0	4.01 (dt, 9.6, 3.0, 1H)
6^″^	59.6	3.70 (d, 3.0, 2H)	59.4	3.70 (d, 3.0, 2H)	59.5	3.70 (d, 3.0, 2H)
7^″^	29.5	2.71 (s, 3H)	29.6	2.70 (s, 3H)	29.6	2.71 (s, 3H)

1^″^′	175.5	–	175.4	–	175.4	–
2^″^′	69.5	4.18 (dd, 9.6, 3.6, 1H)	69.5	4.16 (dd, 9.6, 3.6, 1H)	69.5	4.18 (dd, 9.6, 3.6, 1H)
3^″^′	30.7	2.13 (ddt, 12.6, 3.6, 7.6, 1H)	30.7	2.06 (ddt, 12.6, 3.6, 7.6, 1H)	30.7	2.13 (ddt, 12.6, 3.6, 7.6, 1H)
		1.86 (ddt, 12.6, 9.6, 7.6, 1H)		1.85 (ddt, 12.6, 9.6, 7.6, 1H)		1.86 (ddt, 12.6, 9.6, 7.6, 1H)
4^″^′	36.9	3.07 (t, 7.6, 2H)	36.9	3.06 (t, 7.6, 2H)	36.9	3.07 (t, 7.6, 2H)

From the analysis of HR-qTOF-MS for compound 6′-*N*-acetyl-3^″^-*N*-methyl-amikacin, which was purified as a white amorphous powder, the molecular formula was given as C_2__5_H_4__7_N_5_O_14_: *m*/*z* 642.3193 [M + H]^+^ (calculated for C_2__5_H_4__8_N_5_O_14_^+^, 642.3192), characteristic fragment ions generated by 4,6-glycosidic link cleavages at [M + H - NMeKan]^+^ 467.2350, [1-*N*-AHBA-DOS + H]^+^ 264.1557, [6-*N*-acetyl-aminoGlc + H - H_2_O]^+^ 204.0869, and [NMeKan + H - H_2_O]^+^ 176.0921 (Figures 2A,C). The ^1^H, ^13^C, and HSQC NMR spectra (Supplementary Figures 3A,B,D and Table 1) in D_2_O indicated two carbonyl carbons (δ_C_ 175.4; 174.6), two anomer signals (δ_H_ 5.42/δ_C_ 97.9; δ_H_ 5.07/δ_C_ 97.9), two *N*-methylene signals (δ_H_ 3.34 and 3.10/δ_C_ 39.6; δ_H_ 3.06/δ_C_ 36.9), three *N*-methine signals (δ_H_ 4.01/δ_C_ 48.7; δ_H_ 3.40/δ_C_ 48.4; δ_H_ 3.37/δ_C_ 61.0), an oxygenated methylene signal (δ_H_ 3.70/δ_C_ 59.4), eleven oxygenated methine signals (δ_H_ 3.73/δ_C_ 79.2; δ_H_ 3.76/δ_C_ 66.3; δ_H_ 3.68/δ_C_ 80.1; δ_H_ 3.57/δ_C_ 70.7; δ_H_ 3.68/δ_C_ 72.3; δ_H_ 3.29/δ_C_ 70.7; δ_H_ 3.73/δ_C_ 68.7; δ_H_ 3.79/δ_C_ 72.4; δ_H_ 3.73/δ_C_ 63.5; δ_H_ 4.01/δ_C_ 72.1; δ_H_ 4.16/δ_C_ 69.5), two methylene signals (δ_H_ 2.11 and 1.64/δ_C_ 30.2; δ_H_ 2.06 and 1.85/δ_C_ 30.7), and a methyl signal (δ_H_ 1.90/δ_C_ 21.8), displaying a typical feature of amikacin with an additional acetyl and methyl group. A further interpretation of COSY and HMBC NMR data (Supplementary Figures 3C,E) revealed that amikacin possesses an AHBA, DOS, a 6-aminoGlc, an NMeKan, and an additional acetate. The HMBC correlation from a methyl proton (δ_H_ 1.90) of an acetyl group and protons of *N*-methylene (δ_H_ 3.34 and 3.10) to C-7′ (δ_C_ 174.6) of 6′-*N*-acetyl-amikacin, verified the attachment of an acetyl group on the amine group at C-6′. ^1^H-^13^C correlations from an *N*-methyl proton (δ_H_ 2.70) to C-3^″^ (δ_C_ 61.0) of Kan indicated the attachment of a methyl group on the *N*-methyl at C-3^″^. Therefore, the structure of 6′-*N*-acetyl-3^″^-*N*-methyl-amikacin was confirmed to be a new amikacin analog by 6′-*N*-acylation and 3^″^-*N*-methylation.

6′-*N*-propionyl-3^″^-*N*-methyl-amikacin was enzymatically synthesized and purified as a white powder with the molecular formula C_2__6_H_4__9_N_5_O_14_ according to HR-qTOF-MS: *m*/*z* 656.3354 [M + H]^+^ (calculated for C_2__6_H_5__0_N_5_O_14_^+^, 656.3349); characteristic fragment ions generated by 4,6-glycosidic link cleavages at [M + H - NMeKan]^+^ 481.2510, [1-*N*-AHBA-DOS + H]^+^ 264.1559, [6-*N*-propionyl-aminoGlc + H - H_2_O]^+^ 218.1025, and [NMeKan + H - H_2_O]^+^ 176.0919 (Figures 2A,C). The 1D and HSQC NMR spectra (Supplementary Figures 4A,B,D and Table 1) in D_2_O indicated the presences of two carbonyl signals (δ_C_ 178.6; 175.4), two anomer signals (δ_H_ 5.30/δ_C_ 97.8; δ_H_ 5.07/δ_C_ 97.9), two *N*-methylene signals (δ_H_ 3.43 and 3.40/δ_C_ 39.5; δ_H_ 3.07/δ_C_ 36.9), three *N*-methine signals (δ_H_ 4.01/δ_C_ 48.7; δ_H_ 3.40/δ_C_ 48.4; δ_H_ 3.37/δ_C_ 61.1), an *O*-methylene signal (δ_H_ 3.70/δ_C_ 59.5), 11 oxygenated methine signals (δ_H_ 3.76/δ_C_ 79.3; δ_H_ 3.77/δ_C_ 66.3; δ_H_ 3.75/δ_C_ 80.4; δ_H_ 3.52/δ_C_ 72.0; δ_H_ 3.62/δ_C_ 72.2; δ_H_ 3.21/δ_C_ 70.3; δ_H_ 3.74/δ_C_ 68.7; δ_H_ 3.75/δ_C_ 73.0; δ_H_ 3.71/δ_C_ 63.5; δ_H_ 4.01/δ_C_ 72.0; δ_H_ 4.18/δ_C_ 69.5), three methylene signals (δ_H_ 2.10 and 1.70/δ_C_ 30.1; δ_H_ 2.16/δ_C_ 29.0; δ_H_ 2.13 and 1.86/δ_C_ 30.7), and a methyl signal (δ_H_ 1.01/δ_C_ 8.2). The position of a propionyl group on C-6′ by 6′-*N*-acylation on amikacin was confirmed through HMBC correlations from H-6′ (δ_H_ 3.43 and 3.30), H-8′ (δ_H_ 2.16), and H-9′ (δ_H_ 1.01), respectively, to C-7′ (δ_C_ 178.6) and another ^1^H-^13^C HMBC cross peak from a methyl proton (δ_H_ 2.71) to C-3^″^ (δ_C_ 61.1) of Kan indicated the attachment of a methyl on the amine group at C-3^″^ (Supplementary Figure 4E). Comprehensive analysis of COSY and HMBC revealed an AHBA group, 6′-*N*-propionyl-aminoGlc, and NMeKan on C-1, C-4 and C-6 of DOS, respectively (Supplementary Figures 4C,E). Finally, the structure of 6′-*N*-propionyl-3^″^-*N*-methyl-amikacin was confirmed as a novel amikacin analog by combined analysis of 1D and 2D NMR spectroscopic data.

### Antibacterial Activity and *in vitro* Nephrotoxicity of New Amikacin Analogs

Modification of the C1-amine position on natural AGs has been the best approach for obtaining semisynthetic AG antibiotics with more effective antibacterial activity against resistant bacterial strains compared to their parent drugs, as exemplified by the clinically used semisynthetic AGs shown in Figure 1 (Becker and Cooper, 2013; Krause et al., 2016; Sana and Monack, 2016). The 1-*N*-AHBA group of amikacin has been reported to prevent inactivation in remote susceptible groups, such as 6′-*N*-amine and 3′/4′-*O*-hydroxyl functionalities catalyzed by AAC(6′), APH(3′), and ANT(4′) modifying enzymes, while still preserving most of the antibiotic activity of the parent compound against susceptible strains (Reynolds et al., 1974). Furthermore, it is known that the modification of C6′ or C3^″^-amine positions in natural kanamycins results in increased antibacterial activity against resistant strains (Maeda et al., 1968; Santana et al., 2016). To test whether the selective modification of these two amines of amikacin containing 1-*N*-AHBA could enhance antibacterial activity against amikacin-resistant pathogens, we conducted drug susceptibility tests with amikacin, 3^″^-*N*-methyl-amikacin, 6′-*N*-acetyl-3^″^-*N*-methyl-amikacin, and 6′-*N*-propionyl-3^″^-*N*-methyl-amikacin, following the CLSI guidelines. Amikacin-susceptible and -resistant bacteria (*E. coli* and *P. aeruginosa*) were used to determine the antibacterial spectra of the new analogs (Table 2). Although 3^″^-*N*-methyl-amikacin was less or similarly active against amikacin-susceptible *E. coli* and *P. aeruginosa* compared to amikacin, its antibacterial activity against most of the resistant bacteria was slightly improved. This result was in line with a previous study showing that 3^″^-*N*-guanidino-kanamycin A displayed approximately 2-fold improved activity over a parent drug against *E. coli* expressing AG modifying enzymes and hospital-isolated MRSA strains (Santana et al., 2016). The newly synthesized 6′-*N*-acyl-3^″^-*N*-methylated analogs displayed significantly decreased MIC values against a total of 10 amikacin-resistant pathogens, compared with amikacin in the experiment conducted under the same conditions. Among the compounds tested, 6′-*N*-propionyl-3^″^-*N*-methyl-amikacin showed the strongest antibacterial activity against all tested strains. Based on the obtained data, it can be concluded that additional modification of amikacin by 6′-*N*-acylation and 3^″^-*N*-methylation was effective in circumventing the amikacin resistance of the tested resistant pathogens, although the detailed resistance mechanism of the test strains remains unknown. The importance of 6′-amino group modification in addition to the 1-*N*-AHBA side chain for improved antibacterial activity has also been observed in plazomicin; modifications at the C1 and C6′-amine positions of sisomicin result in the interruption of AG inactivation by clinically relevant AMEs, including AAC(6′), AAC(3), ANT(2^″^), and APH(2^″^) (Aggen et al., 2010; Cox et al., 2018).

**TABLE 2:** Antibacterial activity of amikacin and its analogs.

**Bacterial strains**	**MIC_90_ (μg/mL)**			
	**Amikacin**	**3^″^-*N*-methyl-amikacin**	**6′-*N*-acetyl-3^″^-*N*-methyl-amikacin**	**6′-*N*-propionyl-3^″^-*N*-methyl-amikacin**
*E. coli* ATCC 25922	2–4	8	4	2–4
AREC^a^ P00538	>64	32	16–32	4–8
AREC^a^ P00579	>64	32–64	16	4
AREC^a^ P00650	>64	32	16	4
AREC^a^ P00651	>64	>64	32	4–8
AREC^a^ P00661	>64	32	16	8
*P. aeruginosa* ATCC 27853	8	8	4	4–8
MDRPA^b^ 1–21	>64	>64	16	8
MDRPA^b^ 1–23	>64	32	8	8
MDRPA^b^ 1–67	>64	32	8	4–8
MDRPA^b^ 2–22	>64	>64	16	8
MDRPA^b^ 2–35	>64	32	8	4–8

*^*a*^Antibiotic-resistant *E. coli*.*

*^*b*^Multidrug-resistant *P. aeruginosa*.*

**FIGURE 3:**
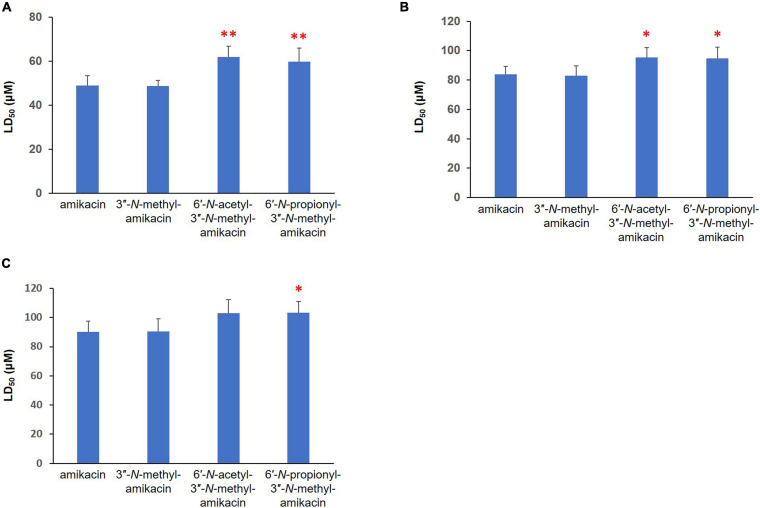
Cytotoxicity of newly synthesized amikacin analogs against three different mammalian renal cell lines. LD_50_ (μM) of amikacin analogs against **(A)** HEK-293, **(B)** LLC-PK1, and **(C)** A-498 cells. Results with ^∗∗^*p* < 0.01 and ^∗^*p* < 0.05 were considered significant within the treatment groups.

The major therapeutic disadvantages of AGs are their high nephrotoxicity and ototoxicity (Chandrika and Garneau-Tsodikova, 2016). To assess the therapeutic potential of the newly synthesized amikacin analogs, we analyzed their *in vitro* toxicity using three mammalian kidney cell lines, HEK-293, LCC-PK1, and A498. In fact, the presence of the 3^″^-*N*-methyl group did not significantly contribute to cytotoxicity. In contrast, the half-maximal lethal concentration (LC_50_) values for 6′-*N*-acetyl-3^″^-*N*-methyl-amikacin and 6′-*N*-propionyl-3^″^-*N*-methyl-amikacin were slightly higher than those of amikacin in the three cell lines tested (Figure 3). Among them, a statistically significant difference in the toxicity profile was observed in human HEK-293 cells: the toxicity of two 6′-*N*-acyl-3^″^-*N*-methylated products was approximately 1.2-fold lower than that of amikacin. These results were consistent with the structure-toxicity relationships observed previously for AGs (Fujisawa et al., 1974; Ban et al., 2019): it was noticed that only the first ring, rather than the structural features of the third ring (Kan moiety), is key to cytotoxicity levels. Additionally, our observations are in agreement with previous results in that the 1, 6′-di-modified AGs exhibited reduced toxicity compared to their parent drug (Chandrika and Garneau-Tsodikova, 2016; Ban et al., 2020a). Hence, it could be suggested that although 3^″^-*N*-methylation slightly improved antibacterial activity, the additional C6′-*N*-acylation is a more effective approach for increasing antibacterial activity against resistant pathogens and reducing the toxicity of amikacin. It is highly likely that the above-described 6′-*N*-acyl-3^″^-*N*-methylated amikacin analogs could be lead compounds for the development of AGs with enhanced antibacterial activity but reduced cytotoxicity.

## Conclusion

The semisynthesis of AGs will certainly continue to play an important role in the development of new antimicrobial drugs against AG-resistant pathogens. In particular, the efficient regiospecific modification method of AG scaffolds is essential for the generation of novel AG analogs with improved therapeutic potential. Our study showed that new amikacin analogs modified at the C6′ and C3^″^-amino groups can be efficiently generated through microbial enzymatic synthesis. Furthermore, the antibacterial activities against amikacin-resistant pathogens and *in vitro* nephrotoxicities were analyzed, and 6′-*N*-acyl-3^″^-*N*-methylated amikacin analogs were found as the potential leads with a superior antibacterial activity and reduced cytotoxicity. Although it is currently unknown how these combined modifications contributed the improved antibacterial activity at molecular level, further molecular docking simulation study would provide valuable insights on the structure-activity relationship of these new analogs. While no new AG therapeutics have yet emerged from microbial enzymatic synthesis, we believe that this approach is an alternative to chemical synthesis and will enable the generation of novel AG analog libraries.

## Data Availability Statement

The original contributions presented in the study are included in the article/Supplementary Material, further inquiries can be directed to the corresponding author/s.

## Author Contributions

YB and YY conceived and designed the experiments. YB, MS, JJ, MK, CK, HR, and EK performed the experiments. YB, MS, and MK wrote the draft manuscript. JP, DL, and YY reviewed and edited the manuscript. All authors contributed to the article and approved the submitted version.

## Conflict of Interest

The authors declare that the research was conducted in the absence of any commercial or financial relationships that could be construed as a potential conflict of interest.

## Publisher’s Note

All claims expressed in this article are solely those of the authors and do not necessarily represent those of their affiliated organizations, or those of the publisher, the editors and the reviewers. Any product that may be evaluated in this article, or claim that may be made by its manufacturer, is not guaranteed or endorsed by the publisher.
