# Supplementary material for: Microbial Enzymatic Synthesis of Amikacin Analogs With Antibacterial Activity Against Multidrug-Resistant Pathogens
Source: Front Microbiol. 2021 Aug 27;12:725916. doi: 10.3389/fmicb.2021.725916 (PMC8430323; doi:10.3389/fmicb.2021.725916)
Supplement: Supplementary file 1 [file Data_Sheet_1.docx]

Microbial enzymatic synthesis of amikacin analogs with antibacterial activity against the multidrug-resistant pathogens

Yeon Hee Ban^1^, Myoung Chong Song^1^, Joong Ho Jeong^1^, Min Seok Kwun^2^, Chang Rae Kim^3^, Hwi So Ryu^3^, Eunji Kim^1^, Je Won Park^3^, Dong Gun Lee^2^, and Yeo Joon Yoon^1*^

^1^ College of Pharmacy, Natural Products Research Institute, Seoul National University, Seoul, South Korea

^2^School of Life Sciences, BK21 FOUR KNU Creative BioResearch Group, College of Natural Sciences, Kyungpook National University, Daegu, South Korea

^3^Department of Integrated Biomedical and Life Sciences, Korea University, Seoul, South Korea

*** Correspondence:**

Yeo Joon Yoon
[yeojoonyoon@snu.ac.kr](mailto:yeojoonyoon@snu.ac.kr)

**
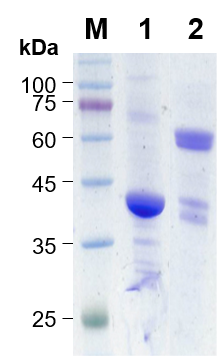
**

Figure S1. Coomassie blue-stained SDS-PAGE gel of purified GenN (40 kDa; Lane 1) and AAC(6′)-APH(2″) (58 kDa; Lane 2).

**
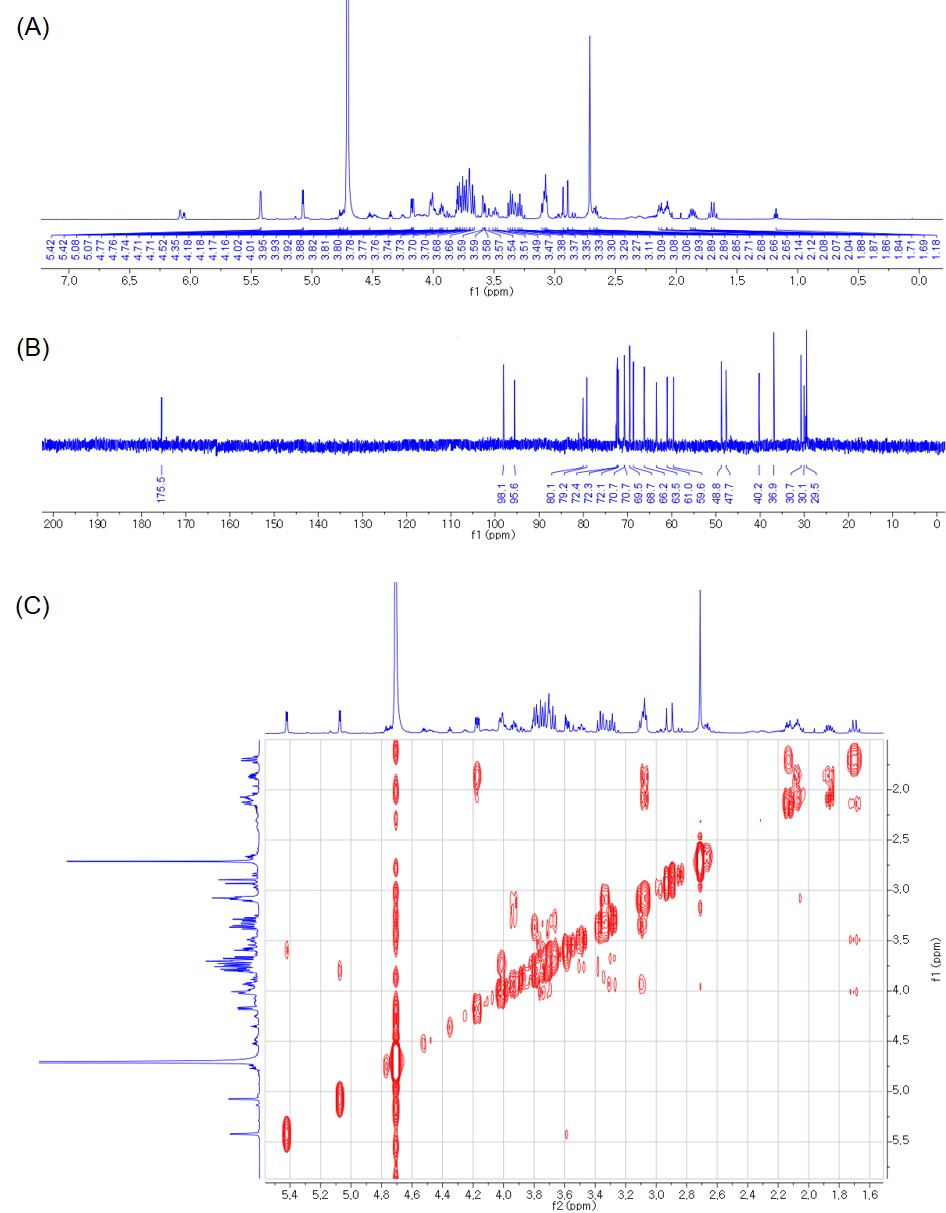
**

**
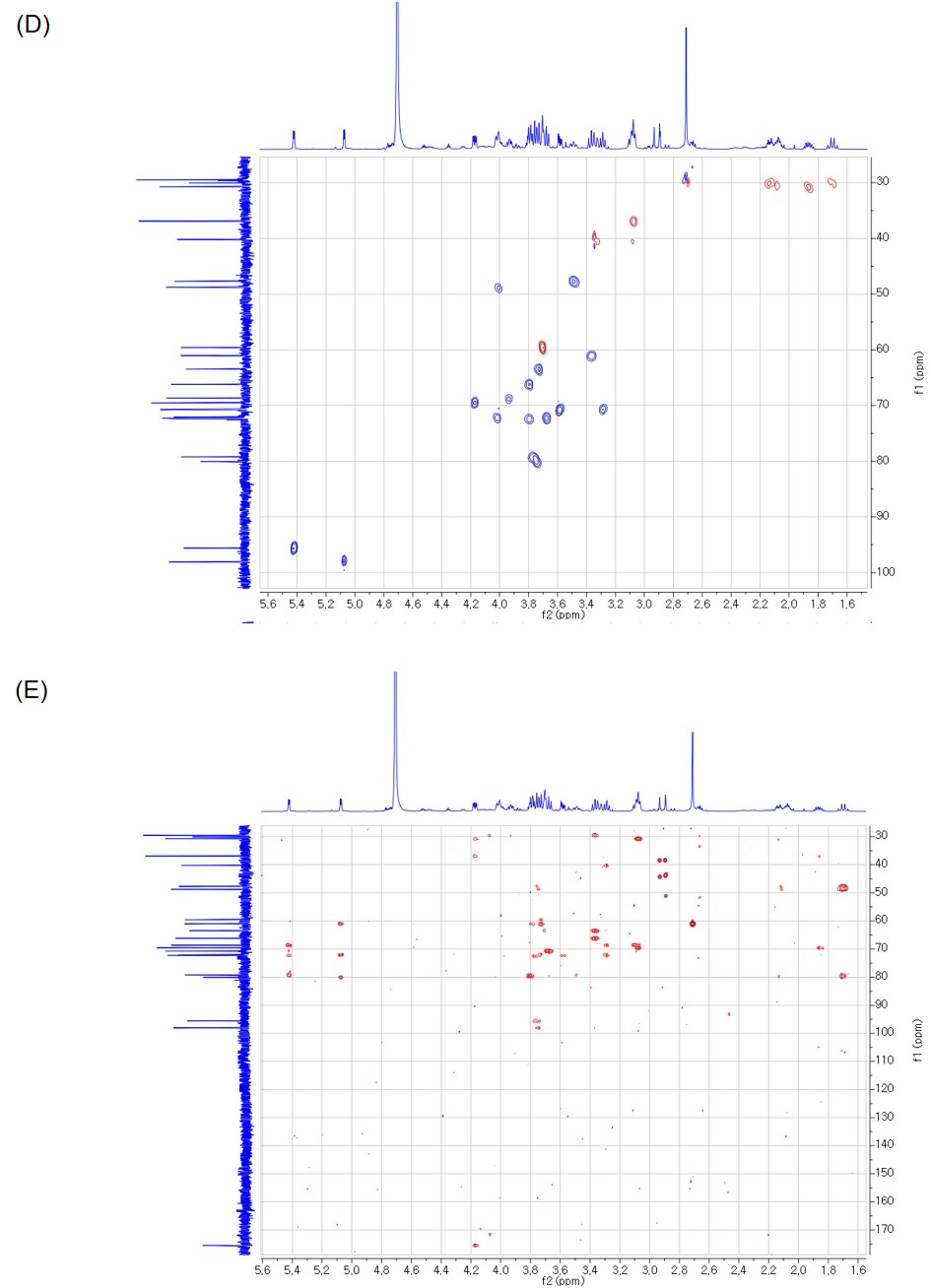
**

Figure S2. The structural determination of 3″-*N*-methyl-amikacin. (A) ^1^H NMR (600 MHz) data (B) ^13^C NMR (150 MHz) data (C) ^1^H-^1^H COSY NMR data (D) ^1^H-^13^C HSQC NMR data (E) ^1^H-^13^C HMBC NMR data

**
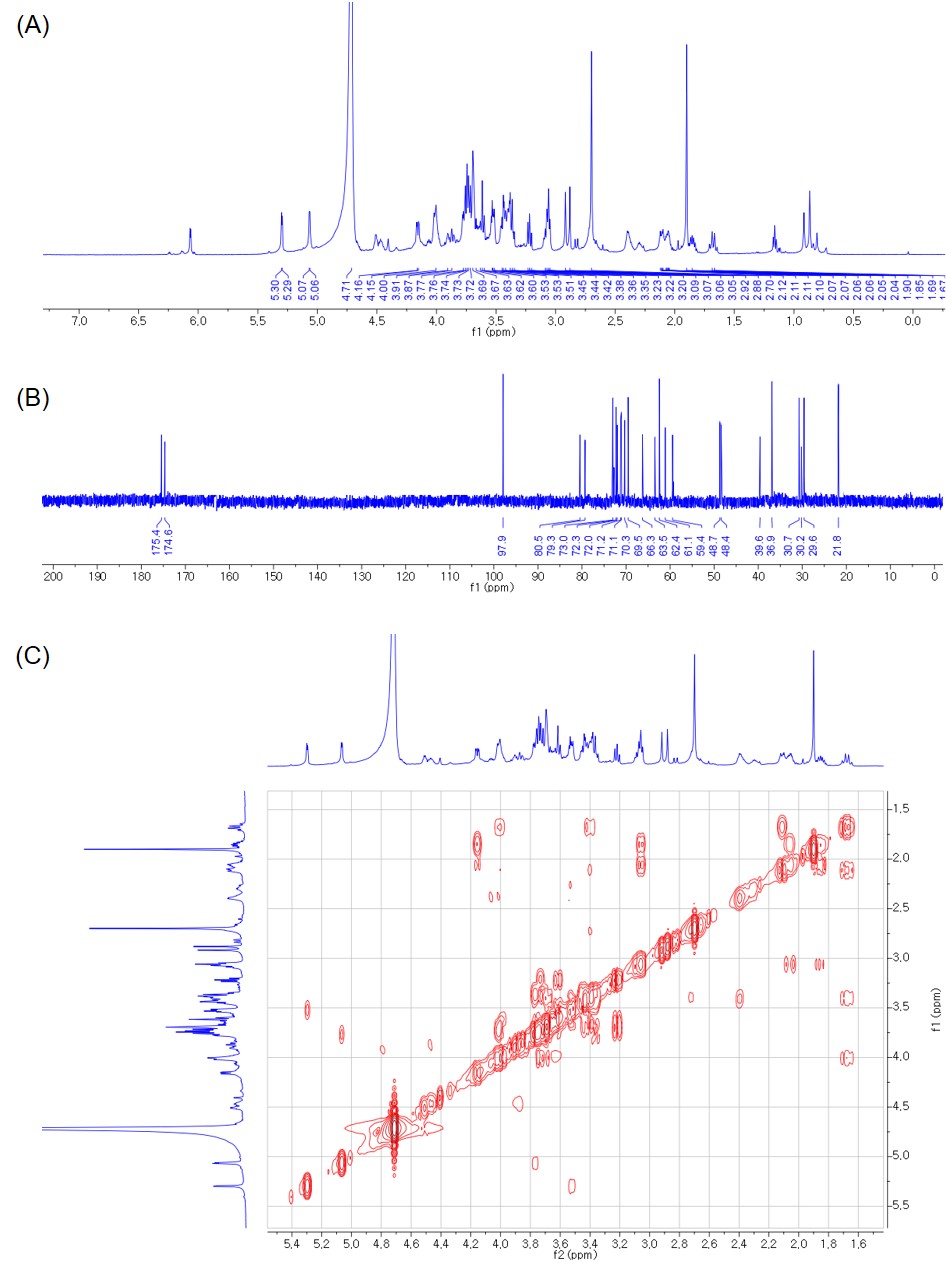
**

**
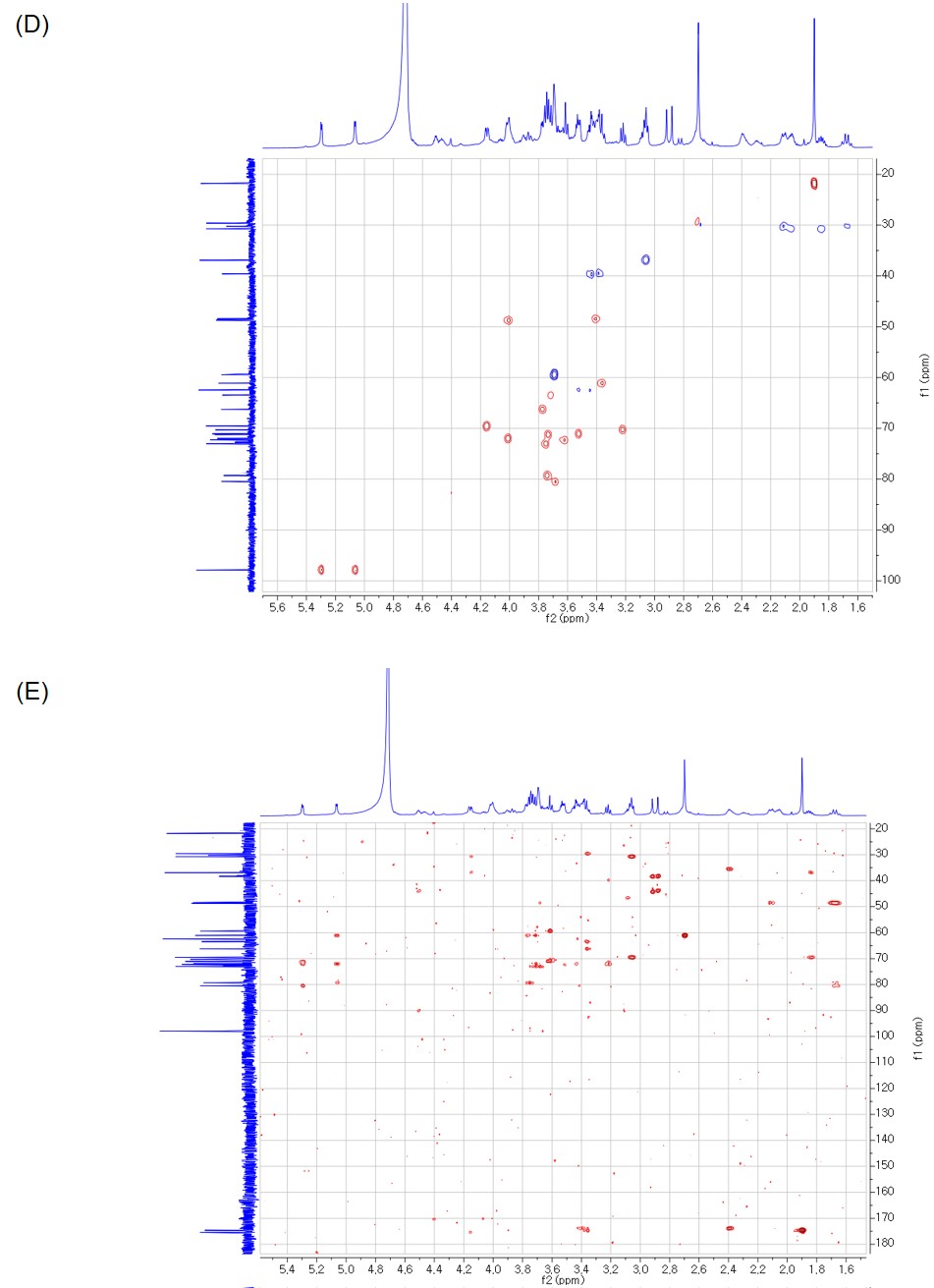
**

Figure S3. The structural determination of 6′-*N*-acetyl-3″-*N*-methyl-amikacin. (A) ^1^H NMR (600 MHz) data (B) ^13^C NMR (150 MHz) data (C) ^1^H-^1^H COSY NMR data (D) ^1^H-^13^C HSQC NMR data (E) ^1^H-^13^C HMBC NMR data

**
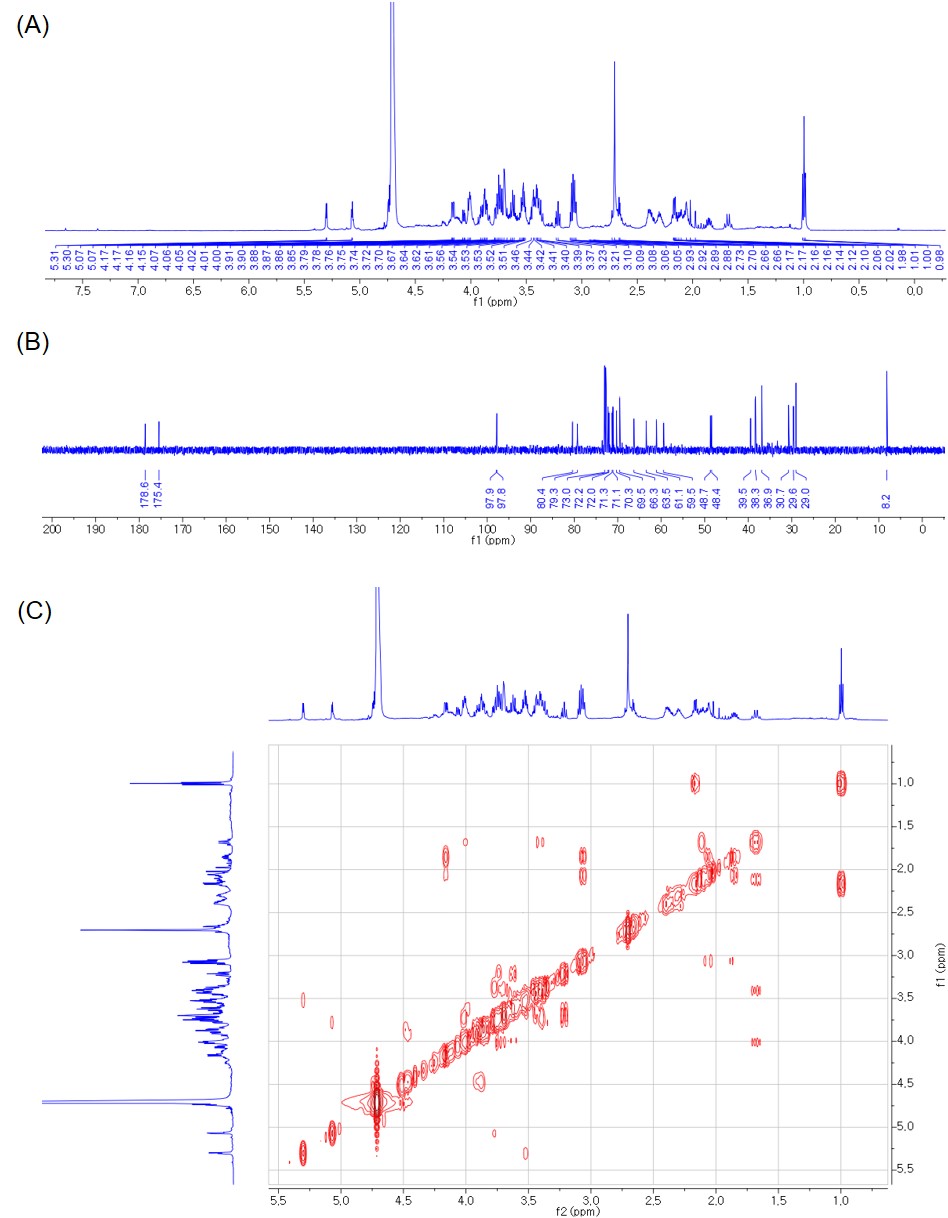
**

**
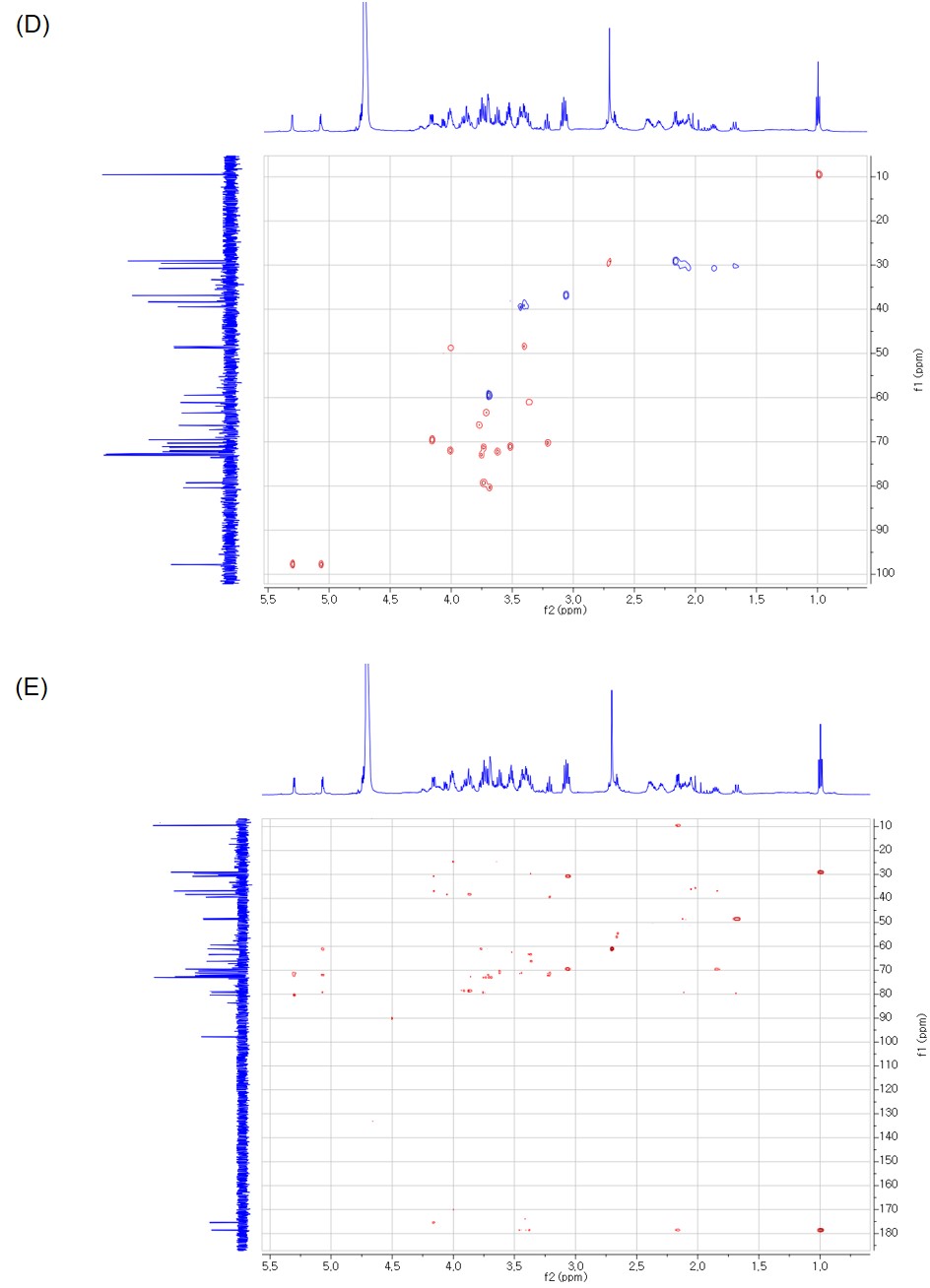
**

Figure S4. The structural determination of 6′-*N*-propionyl-3″-*N*-methyl-amikacin. (A) ^1^H NMR (600 MHz) data (B) ^13^C NMR (150 MHz) data (C) ^1^H-^1^H COSY NMR data (D) ^1^H-^13^C HSQC NMR data (E) ^1^H-^13^C HMBC NMR data
